# De novo transcriptome assembly: a new laccase multigene family from the marine-derived basidiomycete *Peniophora* sp. CBMAI 1063

**DOI:** 10.1186/s13568-017-0526-7

**Published:** 2017-12-20

**Authors:** Igor Vinicius Ramos Otero, Milene Ferro, Maurício Bacci, Henrique Ferreira, Lara Durães Sette

**Affiliations:** 1 Departamento de Bioquímica e Microbiologia, Instituto de Biociências-IB, Universidade Estadual Paulista Júlio de Mesquita Filho-UNESP, 24A, 1515, Rio Claro, SP 13506-900 Brazil; 2 Centro de Estudos de Insetos Sociais, Instituto de Biociências, Universidade Estadual Paulista Júlio de Mesquita Filho, Rio Claro, Brazil

**Keywords:** Marine-derived fungi, Multicopper oxidase, Laccase, Transcriptome

## Abstract

Laccases are multicopper oxidases that are able to catalyze reactions involving a range of substrates, including phenols and amines, and this ability is related to the existence of different laccases. Basidiomycetes usually have more than one gene for laccase, but until now, this feature has not been demonstrated in a marine-derived fungus. *Peniophora* sp. CBMAI 1063 is a basidiomycete fungus isolated from a marine sponge that exhibits the ability to secrete significant amounts of laccase in saline conditions. In the present study, we identified laccase sequences from the transcriptome of *Peniophora* sp. CBMAI 1063 and used them to perform different molecular in silico analyses. The results revealed the presence of at least eight putative genes, which may encode ten different laccases with peptide lengths ranging from 482 to 588 aa and molecular weights ranging from 53.5 to 64.4 kDa. These laccases seem to perform extracellular activities, with the exception of one that may represent an intracellular laccase. The 10 predicted laccases expressed by *Peniophora* sp. CBMAI 1063 in laccase-induced media showed different patterns of N-glycosylation and isoelectric points and are divided into two classes based on the residue associated with the regulation of the redox potential of the enzyme. None of the predicted laccases showed more than 61% similarity to other fungal laccases. Based on the differences among the laccases expressed by *Peniophora* sp. CBMAI 1063, this marine-derived basidiomycete represents a valuable resource with strong potential for biotechnological exploitation.

**Electronic supplementary material:**

The online version of this article (10.1186/s13568-017-0526-7) contains supplementary material, which is available to authorized users.

## Introduction

Laccases (EC 1.10.3.2) are oxidoreductases that are widespread in nature and present in plants, insects, bacteria and fungi, though more expressly in the white rot fungal group (Giardina et al. 2010; Rivera-Hoyos et al. 2013). These enzymes seem to perform different physiological functions, such as lignin synthesis and degradation, spore pigmentation, cell wall elongation and stress defenses (Riva 2006; Giardina et al. 2010).

As a multicopper oxidase, the laccase has an active site with four copper ions. The copper ions are classified per Electron Paramagnetic Resonance (EPR) into three types: type 1—paramagnetic, “blue” ion; type 2—paramagnetic “non-blue” ion, and type 3—diamagnetic pair ion. In general, the type 1 copper ion are linked to two histidine residues, one cysteine residue, and one leucine or phenylalanine residue, while one type 2 and a pair of type 3 ions form a trinuclear cluster linked to eight histidine residues (Claus 2004; Giardina et al. 2010).

Sequence analyses have demonstrated that fungal laccases differ from other multicopper oxidases by a sequence signature corresponding to four conserved regions, namely, L1, L2, L3, and L4. These regions display not only the 12 residues that bind the copper ions but also non-ligand residues, which are involved in the three-dimensional structure of the active site (Kumar et al. 2003; Giardina et al. 2010).

Laccases are known to be capable of accepting a range of substrates such as phenols, amines, and diols, promoting the oxidation of these substrates while reducing molecular oxygen to water (Claus 2004; Riva 2006). Due to these features, laccases have been exploited for biotechnological applications, mainly in the pulp, paper and textile industries and biodegradation of a variety of xenobiotic compounds (Pezzella et al. 2015; Viswanath et al. 2014).

According to Bonugli-Santos et al. (2015), enzymes from marine-derived fungi may have different properties in comparison with that those produced by terrestrial relatives, due to different environmental conditions, such as salinity, temperature, and pressure. Considering the tolerance to saline conditions, these microorganisms are important microbial resources for biotechnological application in bioremediation, including degradation of polycyclic aromatic hydrocarbons (PAH) in ocean and marine sediments (Raghukumar et al. 2006; Passarini et al. 2011). Additionally, a large number of textile processes can generate effluents in saline and alkaline conditions, which can be efficiently decolorized/degraded by fungi from marine environments (Raghukumar et al. 2008; Verma et al. 2010; Chen et al. 2014).

*Peniophora* sp. CBMAI 1063 is a marine-derived basidiomycete that has the ability to express many laccases under saline and non-saline conditions (Bonugli-santos et al. 2010) and biodegrade 94% of the textile dye Reactive Black 5 (RB5) under saline conditions without the production of mutagenic products during the process (Bonugli-Santos et al. 2016). The culture conditions for laccase production by *Peniophora* sp. CBMAI 1063 have been optimized, and a patent have been requested (Bonugli-Santos et al. 2016).

In a previous study, two putative laccase genes from *Peniophora* sp. CBMAI 1063 were suggested based on fragments of approximately 150 bp (Bonugli-santos et al. 2010). However, complete laccase sequences were not available for this fungus. Therefore, the aims of the present study were to obtain the complete laccase sequences of the marine-derived fungus *Peniophora* sp. CBMAI 1063 (after being cultured under optimized conditions for laccase production) and to perform in silico analysis of all sequences in order to compare them with sequences from other basidiomycete fungi.

## Materials and methods

### Microorganism and culture conditions

*Peniophora* sp. CBMAI 1063 was isolated from the Brazilian sponge *Amphimedon viridis* collected in the town of São Sebastião, São Paulo, Brazil (Menezes et al. 2010) and taxonomically identified as reported by Bonugli-Santos et al. (2010). The strain is being maintained using different preservation methods at the Brazilian Collection of Environmental and Industrial Microorganisms—CBMAI (UNICAMP, SP, Brazil) and at the UNESP Central of Microbial Resources—CRM-UNESP (UNESP, SP, Brazil).

The fungus was cultivated for 7 days at 28 °C in a laccase expression-optimized medium (patent request deposited at Instituto Nacional de Propriedade Industrial—INPI under the number BR102014008502) composed of yeast extract (0.2%), bacteriological peptone (0.27%), malt extract (0.14%), d-glucose (0.27%), and artificial sea water adapted from Kester et al. (1967), ASW: 0.704% MgCl_2_, 0.098% CaCl_2_, 0.001% SrCl_2_, 1.555% NaCl, 0.261% Na_2_SO_4_, 0.044% KCL, 0.013% NaHCO_3_, 0.006% KBr and 0.002% H_3_BO_3_, supplemented with 2 mM CuSO_4_ as laccase inductor.

### RNA extraction and sequencing

Total RNA from *Peniophora* sp. CBMAI 1063 was extracted using the RNeasyPlant Mini Kit (QIAGEN), according to manufacturer’s protocol. The integrity of the RNA was examined by 0.7% agarose gel electrophoresis, and the concentration was estimated using a NanoDrop 2000 spectrophotometer. The cDNA library construction and sequencing were performed in 1/3 lane using the Illumina Hiseq 2000 platform, paired-end 2 × 100 bp according to the manufacturer’s protocol from MACROGEN (Seoul, South Korea).

### De novo assembly and functional annotation

The reads quality was assessed using the FastQC (Andrews 2010) program. Trimming of reads was performed with trimmomatic (Bolger et al. 2014) using the minimum quality filtering (Phred 20) functionality of this tool with a sliding window, which scans through reads from the 5′ end and removes subsequent bases from the 3′ end once the average quality score within the window drops below a user-specified value (minimum size 50 bp).

De novo assembly was performed using Trinity (Grabherr et al. 2011) with the parameter ‘min_kmer_cov 2’ following the method described by Haas et al. (2013). The use of this parameter increases the stringency for reads being assembled together (Chapman 2015). Thus, only the kmers that occur more than once are considered for the contigs, and the default is that all kmers are considered (Johnson 2015). We prepared a set of non-redundant contigs (unigenes) by selecting only the longest contigs among the isoforms.

The functional annotation was performed using the Blast2GO PRO version (Gotz et al. 2008) that describes the unigenes using the BLASTx algorithm (Altschul et al. 1990) with an E-value threshold of 1.0E−3 against the NCBI non-redundant (Nr) database to identify protein domains with the InterProScan (Zdobnov and Apweiler 2001) tool and assign the gene ontology (GO) and enzyme commission (EC) terms. Annotations using Blast2GO were conducted with 1.0E−6 as the E-value hit filter, 55 as the annotation cut-off and 5 as the GO weight.

### Analysis of the laccase sequences

Sequences that returned from the Nr database as laccase were submitted to ORF finder (https://www.ncbi.nlm.nih.gov/orffinder/). The ORFs with the largest lengths were selected, and the translated products were aligned using ClustalW (Bioedit 7.0). After the alignments, a search of the conserved regions L1, L2, L3, and L4 was performed according to Kumar et al. (2003), in order to obtain only true laccases.

GeneRunner 5.0 was used to determine the size length of the coding sequence and the peptide chain. The peptide composition, molecular weight and isoelectric point (pI) were determined using ProtParam (http://web.expasy.org/protparam/) (Gasteiger et al. 2005). The similarity analysis with other fungal laccases and multicopper oxidases was performed using MegAlign (DNASTAR 14.1.0.115) (Eggert et al. 1998); the DNA and protein sequences from other organisms used in this analysis were obtained from the NCBI database. SignalP 4.1 (http://www.cbs.dtu.dk/services/SignalP/) was used, with SignalP 3.0 default, to recognize signal peptide for extracellular activity and predict cleavage sites for Peptidase I (Bendtsen et al. 2004; Petersen et al. 2011). The prediction of N-glycosylation sites was performed with the NetNGlyc 1.0 server (http://www.cbs.dtu.dk/services/NetNGlyc/) (Vite-Vallejo et al. 2009), and the results were confirmed using GlicoEP (http://www.imtech.res.in/raghava/glycoep/submit.html) (Chauhan et al. 2013). The phylogenetic analysis was performed using MEGA 6.0 (Tamura et al. 2013). The distances were calculated using the neighbor-joining method and a bootstrap with 1000 pseudoreplications (Felsenstein 1985; Saitou and Nei 1987).

### Accession numbers

The raw sequences data from the *Peniophora* sp. CBMAI 1063 transcriptome are available at Short Read Archives (SRA) GenBank database, deposited under the Accession Number No. SRR5799684 (BioProject: PRJNA392894). Putative laccase genes were also deposited in GenBank under the followed Accession Numbers: Lcc1 no. MF176136; Lcc2 no. MF176137; Lcc3 no. MF176138; Lcc3B no. MF176139; Lcc4 no. MF176140; Lcc5 no. MF176141; Lcc5B no. MF176142; Lcc6 no. MF176143; Lcc7 no. MF176144; Lcc8 no. MF176145.

### Experimental in vitro validation

Two of the laccase sequences obtained from *Peniophora* sp. CBMAI was selected and cloned in *Escherichia coli*. The specific primers to each one of the sequences were designed using GeneRunner 5.0 (Additional file 1: Table S1). A first RT-PCR was performed according to the manufacture’s protocol (RevertAid H Minus Reverse Transcriptase—Thermo Scientific) with the oligo-dT primer to reverse transcribe the total mRNA of the fungus to cDNA. Afterward, laccase sequences amplification was performed by touchdown PCR using the designed primers. PCR conditions were as follows: 2 min of initial denaturation at 94 °C, followed by a touchdown step of 30 s from 74 °C to 62 °C (due to the difference of the forward and reverse annealing primers), 35 cycles of 30 s at 94 °C and 30 s at 62 °C and a final extension step of 5 min at 72 °C. PCR products were detected by 0.7% agarose gel electrophoresis, purified using the GeneJET gel Extraction Kit (Thermo Scientific) according to manufacturer’s protocol, and ligated into the pJET 1.2 cloning vector (Thermo Scientific). The *E. coli* DH10B strain was used as the cloning host, and six clones were selected to be sequenced using the Sanger method at MACROGEN (Seoul, South Korea).

## Results

### Transcriptome annotation

Sequencing generated 11,005,713,864 total bases and 108,967,464 reads. Trinity de novo assembly generated 36,981 contigs (including isoforms) with an average length of 1552 bp. A total of 16,663 non-redundant contigs (unigenes) were selected. The Blast2GO PRO results showed that 10,649 unigenes had significant similarity to known proteins in NCBI-Nr, 8367 had significant similarity with the InterPro domains and 3838 unigenes presented at least one GO term.

Among the unigenes submitted to the NR protein database (NCBI), 43% presented high similarity to other sequences, and all the top hits were related to terrestrial basidiomycetes. The *Heterobasidion irregulare* and *Stereum hirsutum* sequences presented the highest similarities to the *Peniophora* sp. CBMAI 1063 unigenes (Additional file 1: Figure S1).

The unigenes (3838) assigned to GO terms level 2 were classified into 39 functional groups belonging to three categories: molecular functions, biological process, and cellular process. Within molecular functions, “catalytic activity” and “binding” represented the most abundant subcategories with 1260 unigenes and 972 unigenes, respectively, while “metabolic processes”, “cellular processes”, and “single-organism processes” were the most representative subcategories in biological processes, with 1056, 956, and 757 unigenes, respectively. Finally, “cell”, with 471 unigenes, was the most representative functional group in cellular processes (Additional file 1: Figure S2).

Among the enzymes expressed by the fungus *Peniophora* sp. CBMAI 1063, transferases, with 180 unigenes, comprised the most representative group, followed by hydrolases with 169 unigenes and oxidoreductases with 111 unigenes.

### Analysis and characterization of the laccase transcripts

Forty-seven sequences of laccase were found in the transcriptome. Among them, 13 presented all four conserved regions that are characteristic of known laccases. All putative laccases showed similarity to laccases from other basidiomycetes and multicopper oxidases from another *Peniophora* species. However, three putative laccase sequences were likely pseudogenes lacking a stop codon (comp15071_c0_seq1 and comp15071_c0_seq4) or presenting a stop codon interposed within the coding sequence (comp8257_c0_seq1).

**Fig. 1:**
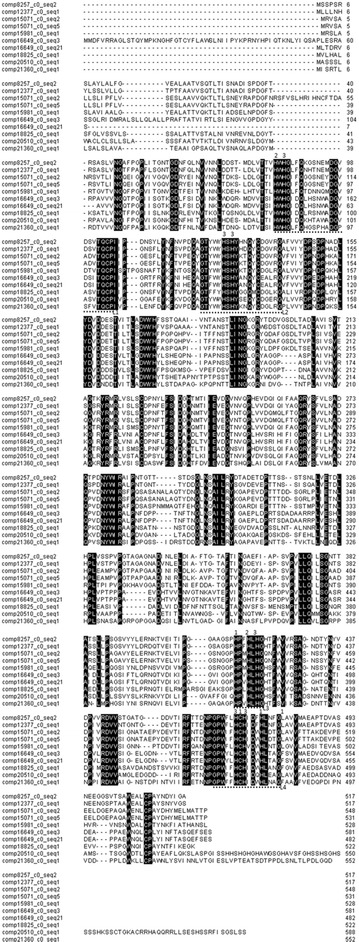
Predicted amino acid sequence alignments of all 10 putative laccases from *Peniophora* sp. CBMAI 1063. Amino acids with 100% matches are highlighted in black. Numbers above the amino acids indicate that they are copper ion-bound. Dots below the amino acids indicate conserved regions in the fungal laccases (L1, L2, L3 and L4)

Figure 1 shows the alignment of the 10 putative laccase sequences containing the four conserved regions and copper ligand sites. The sequences contained 1449–1767 bp, and all of them presented high GC contents, with the percentage ranging from 52.2 to 58.9%. The predicted polypeptide chain varied between 482 and 588 aa with peptide weights ranging from 53.5 to 64.4 kDa. The laccases found in the transcriptome represent extracellular laccases, with the exception of Lcc5B, which did not show a peptide cleavage site and seemed to be an intracellular enzyme. Table 1 shows the complete characterization with base pair length, peptide chain length, molecular weight, GC content, cleavage site for Peptidase I and theoretical pI, of all 10 putative laccases.

**Table 1:** Complete characterization of laccase-codifying transcripts from *Peniophora* sp. CBMAI 1063

Sequence	Laccase	Length (bp)	Peptide chain (aa)	Mol. weight (kDa.)	GC content (%)	Cleavage site	Theoretical pI
comp8257_c0_seq2	Lcc1	1554	517	55.5	58.1	20–21	4.25
comp12377_c0_seq1	Lcc2	1554	517	55.9	53.9	22–23	4.21
comp15071_c0_seq2	Lcc3	1647	548	60.5	58.6	19–20	4.68
comp15071_c0_seq5	Lcc3 B	1596	531	58.5	58.9	19–20	4.51
comp15981_c0_seq1	Lcc4	1587	528	58	53.6	22–23	5.51
comp16649_c0_seq3	Lcc5	1545	514	57	52.4	17–18	5.61
comp16649_c0_seq21	Lcc5 B	1449	482	53.5	52.2	No	5.49
comp18825_c0_seq1	Lcc6	1569	522	56.6	53.9	21–22	4.92
comp20510_c0_seq1	Lcc7	1767	588	64.4	55.2	16–17	6.12
comp21360_c0_seq1	Lcc8	1659	552	59.9	57.6	18–19	4.79

Amino acid sequence analysis revealed that two types of laccases were expressed by *Peniophora* sp. CBMAI 1063 based on a variable copper type 1 ligand, which is related to the influence in the reduction–oxidation potential. At the variable position, six sequences contained leucine and four contained phenylalanine.

Except for Lcc5B, all laccases exhibited approximately four to ten sites that could be N-glycosylated; some sites were common to more than one sequence, and other sites were similar to those found in laccases from different fungi (Table 2).

**Table 2:** N-glycosylation site prediction of the 10 putative laccases from *Peniophora* sp. CBMAI

Laccase	N-glycosylation sites									
Lcc1	*185*	*380*	398	*431*						
Lcc2	47	90	115	*185*	239 ^4^	*289*	314	*380*	*431*	497
Lcc3	39	56	131	201	246	255	420	453	471	
Lcc3B	39	114	*184*	229 ^1,3^	238 ^2,3^	403	436	*454*		
Lcc4	187	232	322	388	439	458 ^2^				
Lcc5	248	351	458 ^2^	513	570					
Lcc5B^a^	–	–	–	–	–	–	–	–	–	–
Lcc6	48	67	117	*289*	290	349	*359*	*396*	*433*	*454*
Lcc7	36	*184*	291	351	*359*					
Lcc8	*185*	312	331	350	363	*396*	*433*	450	543	

^a^Lcc5B does not present peptide signal and may not pass through N-glycosylation process. Italic numbers: site similar to other laccases from *Peniophora* sp. CBMAI 1063. Underlined numbers: site similar to laccases from other basidiomycetes

^1^Similar to Lcc3-1 from *Pycnoporus cinnabarinus* (Accession Number AF025481)

^2^Similar to Lcc4 from *Lentinus* sp. (Accession Number KF836751)

^3^Similar to Lcc1 from *Trametes villosa* (Accession Number L49376)

^4^Similar to pox1 from *Pleorotus ostreatus* (Accession Number Z34847)

The putative laccases of *Peniophora* sp. CBMAI 1063 showed high similarity (80–93%) to the multicopper oxidases found in the genome of *Peniophora* sp. (Nagy et al. 2015) but presented low similarity (below 60%) to other fungal laccases (Table 3).

**Table 3:** Levels of similarity among the laccases from *Peniophora* sp. CBMAI 1063, other laccases and multicopper oxidases

Organism/Lcc or MCO	% similarity among laccases and other MCOs																													
	1	2	3	4	5	6	7	8	9	10	11	12	13	14	15	16	17	18	19	20	21	22	23	24	25	26	27	28	29	30
1. P. sp. 1063 Lcc1		81.2	60.2	60.2	61.3	54.9	56.7	55.1	54.6	53.4	56.4	61.5	54.3	89.1	82.2	58.9	55.2	54.3	54.9	60.1	58.1	59.7	56.9	60.8	58.2	58.4	59.0	60.9	61.2	56.2
2. P. sp. 1063 Lcc2			61.0	61.0	60.9	54.7	56.3	53.7	54.8	50.6	55.6	62.5	51.5	80.2	83.2	60.1	55.6	53.3	54.5	58.1	59.4	58.9	55.3	57.6	56.6	55.0	56.6	59.5	59.6	56.8
3. P. sp. 1063 Lcc3				100.0	61.1	51.1	52.1	50.3	50.2	49.6	53.8	62.4	49.5	59.3	60.5	93.6	49.6	48.7	51.7	54.1	52.7	52.3	54.9	53.9	53.1	48.5	49.9	51.4	56.0	54.0
4. P. sp. 1063 Lcc3 B					61.1	51.0	52.1	50.3	50.2	49.6	53.8	62.4	49.5	59.3	60.5	93.6	49.6	48.7	51.7	54.1	52.7	52.3	54.9	53.9	53.1	48.5	49.9	51.4	56.0	54.0
5. P. sp. 1063 Lcc4						53.3	54.6	52.7	54.3	51.1	55.0	83.9	50.3	60.9	59.8	58.9	52.7	53.8	53.9	53.5	56.0	53.2	53.1	55.0	51.7	49.2	52.8	53.5	55.8	57.2
6. P. sp. 1063 Lcc5							99.0	52.0	55.8	50.1	80.7	55.1	52.2	53.8	54.3	50.1	56.8	50.6	53.3	53.0	53.8	51.7	55.5	53.1	52.6	52.3	53.0	56.9	54.6	53.8
7. P. sp. 1063 Lcc5 B								53.6	56.0	51.3	81.1	56.1	52.9	55.8	55.7	51.4	57.2	51.4	55.0	54.5	55.8	53.3	55.7	54.2	53.4	53.9	53.9	57.8	56.1	54.6
8. P. sp. 1063 Lcc6									52.5	49.2	52.8	52.5	81.9	54.2	53.1	49.0	52.1	50.8	50.4	52.4	50.8	50.4	49.7	50.1	48.2	49.6	49.2	51.5	53.8	49.3
9. P. sp. 1063 Lcc7										48.6	58.0	54.1	52.9	54.9	52.8	50.5	83.8	50.0	50.7	48.4	55.4	52.6	53.1	48.9	51.1	49.8	51.3	52.1	55.1	50.9
10. P. sp. 1063 Lcc8											50.3	51.7	48.6	52.5	50.8	49.8	47.9	81.4	54.7	50.9	55.5	52.9	53.3	50.8	52.5	51.9	53.3	54.8	54.2	53.3
11. P. sp. MCO 1												55.8	52.1	55.7	53.8	52.4	58.8	51.3	54.9	53.8	55.9	53.2	57.1	53.7	53.7	52.8	53.4	56.4	56.4	53.8
12. P. sp. MCO 2													50.1	61.6	61.0	61.0	54.3	53.8	54.9	54.6	56.6	54.7	55.3	55.2	52.7	50.4	53.4	54.5	57.2	59.0
13. P. sp. MCO 3														53.2	52.9	48.4	53.5	49.6	50.0	51.6	50.0	51.0	48.9	48.7	48.0	48.2	49.0	51.1	53.4	47.0
14. P. sp. MCO 4															81.2	58.3	54.9	54.0	53.6	58.6	59.2	59.4	56.4	60.1	58.5	57.7	58.5	60.4	61.3	55.9
15. P. sp. MCO 5																59.8	53.8	53.7	52.9	57.3	57.5	58.3	54.1	57.0	56.6	55.6	56.8	58.3	58.4	55.6
16. P. sp. MCO 6																	49.3	48.2	51.4	54.0	52.2	52.3	54.2	53.4	52.2	48.8	49.4	51.1	55.3	53.7
17. P. sp. MCO 7																		48.7	51.7	49.8	53.1	51.2	54.1	48.8	51.3	49.5	49.8	52.2	55.9	51.3
18. P. sp. MCO 8																			56.7	52.4	56.9	54.4	54.3	53.0	53.5	53.9	55.2	56.2	55.4	54.5
19. H. i. Lcc																				58.5	60.3	59.7	58.5	58.1	56.3	57.9	58.7	61.1	59.8	62.7
20. C. co Lcc																					60.1	63.9	67.5	60.7	64.1	62.7	64.6	67.1	80.1	62.4
21. H. c. Lcc																						65.0	57.5	62.1	58.0	57.2	58.5	61.5	61.1	59.2
22. S. h. Lcc																							60.3	59.9	62.4	58.2	62.2	64.6	63.2	59.6
23. C. ci. Lcc																								60.4	64.1	60.2	61.3	65.5	66.5	62.2
24. S. m. Lcc																									60.2	62.8	66.3	70.8	66.7	59.0
25. P. o. Lcc																										60.4	63.0	66.6	65.8	61.3
26. D. s. Lcc																											73.6	66.3	60.8	58.3
27. C. g. Lcc																												68.9	63.3	60.0
28. M. g. Lcc																													68.7	62.2
29. L. sp. Lcc																														63.1
30. M. c. Lcc																														

Lcc, laccase; MCO, multicopper oxidase; P. sp. 1063, *Peniophora* sp. CBMAI 1063; P. sp., *Peniophora* sp.; H. i., *Heterobasidion irregulare*; C. co. *Coprinus comatus*; H. c., *Hericium coralloides*; S. h., *Stereum hirsutum*; C. ci., *Coprinopsis cinerea*; S. m., *Steccherinum murashkinsky*; P. o., *Pleorotus ostreatus*; D. s., *Dichomitus squalens*; C. g., *Coriolopsis gallica*; M. g., *Meripilus giganteus*; L. sp., *Leucoagaricus* sp.; M. c., *Mycena chlorophos*

**Fig. 2:**
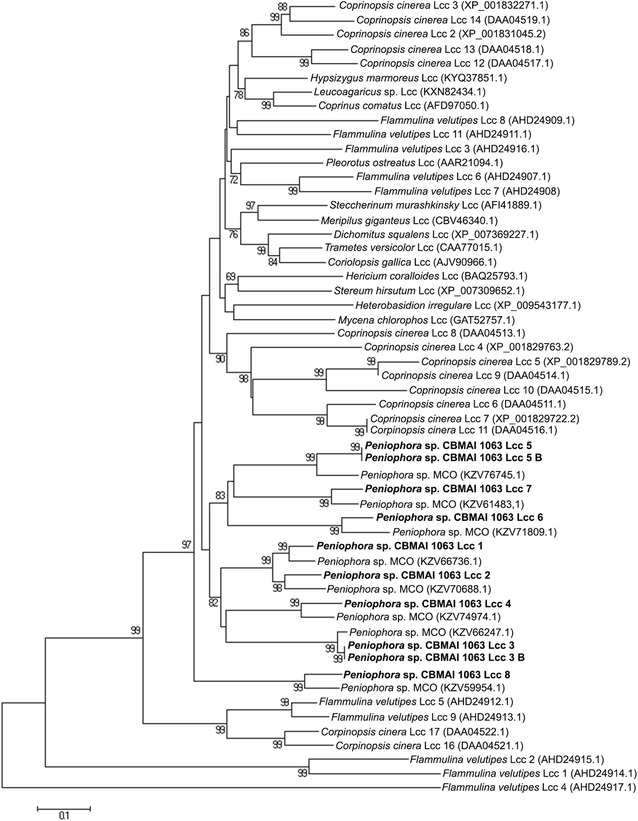
Phylogenetic tree based on the Lcc1 to Lcc8 sequences, other basidiomycete laccases and putative multicopper oxidases from *Peniophora* sp. (see Nagy et al. 2015). Two complete laccase families from *Flammulina velutipes* and *Coprinopsis cinerea* are presented in the tree. The scale bar indicates a distance equivalent to 0.1 amino acid substitutions per site

Data from phylogenetic analysis suggest a gene family with eight different genes, due to the formation of eight different clades involving all 10 putative laccases. Furthermore, according to the tree (Fig. 2) Lcc3 and Lcc3B should be considered identical laccases, as well as Lcc5 and Lcc5B. However, the amino acid analyses revealed that short insertions differentiated these laccases. This result leads to a conclusion that the enzymes Lcc3/Lcc3B and Lcc5/Lcc5B may arises from alternative splicing of the genes *Lcc*3 and *Lcc*5, respectively.

The gene family from *Peniophora* sp. CBMAI 1063 did not group with other fungal laccases and formed a separate cluster that included seven multicopper oxidases from *Peniophora* sp. However, Lcc8 grouped in a separated clade with only one other multicopper oxidase (Fig. 2).

### In vitro validation

**Fig. 3:**
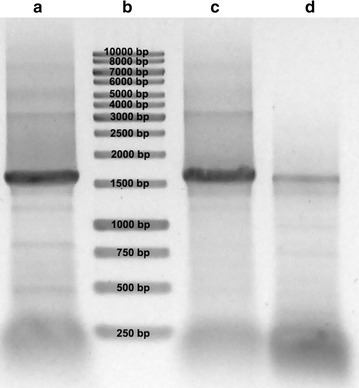
PCR of comp15071_c0_seq5 (Lcc5B) with three different polymerases. Bands with the length of approximately 1.500 bp correspond to the predicted size of the sequence. **a** amplification with Pfu platinum DNA polymerase (Thermo Scientific), **b** 1-kb ladder (Promega), **c** amplification with Taq DNA polymerase (Promega), **d** amplification with Phusion DNA polymerase (Thermo Scientific)

The most expressed laccase, according with FPKM factor (data not shown), did not present stop codon in its sequence and was considered as pseudogene thus two other laccases were selected based on high similarity with the most expressed laccase also using FPKM factor (data not shown): Lcc3 and Lcc3B. Although amplifications showed sequences with the expected size, it was not possible to clone and sequence fragments from Lcc3. Six clones from Lcc3B were sequenced and compared with the sequence obtained in the transcriptome. After amplification, the Lcc3B sequence showed approximately 1500-bp band in the agarose gel (Fig. 3). The sequence of the cloned fragment was 100% identical to the sequence of Comp15071_c0_seq5 from transcriptome (Table 1).

## Discussion

According to Giardina et al. (2010), most of the fungal laccases are glycoproteins with extracellular activity and molecular weights ranging from 60 to 70 kDa. The majority of putative laccases expressed by *Peniophora* sp. CBMAI 1063 had molecular weights near or higher than 60 kDa, corresponding to extracellular enzymes. However, Lcc5B seems to play an intracellular role. The existence of an intracellular laccase has already been reported in *Trametes versicolor* (Schlosser et al. 1997), *Pleurotus ostreatus* (Palmieri et al. 2000), and *Flammulina velutipes* (Wang et al. 2015) and may be related in these organisms to the low molecular weight phenol oxidation, cell division and elongation processes (Baldrian 2006; Wang et al. 2015).

Eggert et al. (1998), suggested three classes of laccases based on the variable residues that bind the copper type 1 ion (molecular analysis). Class 1 has methionine, class 2 has leucine, and class 3 has phenylalanine at this position. According to this classification, six putative laccases from *Peniophora* sp. CBMAI 1063 belong to class 2, while four laccases belong to class 3. Site-directed mutagenesis of the residues that occupy this position seems to interfere with the redox potential due to the alteration in the coordination of the T1 copper ion (Xu et al. 1996, 1999). The theoretical pI prediction ranged from 4.21 to 6.12, based on differences found in the amino acid compositions of the putative laccases. These results were expected, and together with other results, these data reinforce the idea that the laccases from *Peniophora* sp. CBMAI 1063 may act on different substrates under acidic conditions.

Laccases generally have an expressive glycosidic portion, which may represent approximately 10–45% of the total mass (Claus 2004). Mannose seems to be the most representative carbohydrate in fungal laccases, and in association with other sugars, mannose constitutes the glycosidic moiety. The glycosidic portion guarantee the stability in the enzyme, minimize protease susceptibility, signal extracellular activity, and influence redox potential (Dwivedi et al. 2011; Vite-Vallejo et al. 2009). In the present study, different N-glycosylation sites were predicted for nine putative laccases, which presented among 4–10 possible sites. However, some sites were too close to each other to allow simultaneous glycosylation. In this sense, sites that were homologous to those found in other fungal laccases could in fact be glycosylated.

The occurrence of multiple laccase genes seems to be recurrent in many basidiomycete genomes. The first laccase gene family was reported in *Agaricus bisporus*, which exhibited two different laccase genes in the same chromosome (Giardina et al. 2010). Afterward, other gene families were reported in *Trametes villosa*, and *F. velutipes* with 13 and 11 genes (Wang et al. 2015), respectively, and *Coprinopsis cinerea* with 17 genes (Kilaru et al. 2006). Representatives of the genus *Peniophora* were also reported as laccase producers with at least five different laccase isoenzymes (Niku-Paavola et al. 2004).

However, there were no data in the consulted literature related to the presence of a multiple-laccase gene family from a marine-derived basidiomycete. In the present study, 8 putative laccase genes with 10 possible enzyme products were found in the transcriptome of *Peniophora* sp. CBMAI 1063.

According to Valderrama et al. (2003), most of the fungal laccase multigene families arise from duplication events. If the duplication occurs after the last speciation, laccase genes from the same family groups will be in the same clade in a neighbor-joining analysis. On the other hand, if the duplication event occurs before the last speciation, these genes may assemble with other laccase families. These evolutionary relationships lead to a conclusion that the majority of the laccase genes in *Peniophora* sp. CBMAI 1063 arose from the last speciation, except for Lcc8, which may have arisen from an earlier duplication event. Although all laccases from *Peniophora* sp. CBMAI 1063 grouped with the multicopper oxidases from *Peniophora* sp., the sequence analysis revealed that these multicopper oxidases also exhibited the laccase signature (data not shown).

Different laccase genes in a single genome suggest that the enzymes play different physiological functions in the organism. Laccases have been associated with fruiting body development, spore pigmentation, pathogenesis, cell elongation, the duplication process, the stress response, and lignin bioconversion (Giardina et al. 2010; Rivera-Hoyos et al. 2013). Neighbor-joining analysis allowed a prediction laccase function using its similarity to other identified genes. However, none of the putative genes grouped with a well-identified gene, so further studies are needed to unveil all of the functions of the laccase isoenzymes in the *Peniophora* sp. CBMAI 1063 physiology.

In optimized conditions, *Peniophora* sp. CBMAI 1063 was able to express at least 10 different laccases based on peptide chain length, peptide composition, molecular weight, glycosylation pattern, and cellular activity site. It is important to highlight that in a previous study carried out by our research group, the marine-derived fungus *Peniophora* sp. CBMAI 1063, after has being cultured in the optimized conditions for laccase production (the same conditions used in the present study), was able to produce great amounts of laccase only in the presence of artificial seawater (saline condition) and copper sulfate (data not published yet).

Considering the marine origins of the new putative laccases, it is expected a high-salt tolerance from these enzymes, which represents a great potential to apply them in industrial and/or environmental processes performed under saline conditions. To this end, studies related to the expression and characterization of these enzymes, involving genetic improvement and heterologous expression, should be performed.

## Additional file

**Additional file 1.** **Table S1.** Specific primers designed for Comp15071_c0_seq5 with tails to bind amplification products in the cloning vector. **Figure S1.** Species distribution of all homologous unigenes. **Figure S2.** Gene ontology (GO) classification of assembled unigenes (Level 2). GO terms were distributed into three ontologies: molecular functions—blue bars; biological process—red bars; and cellular component—green bars.

## Abbreviations

aa: amino acid
bp: base pair
CBMAI: Coleção Brasileira de Micro-organismos de Ambiente e Indústria
cDNA: complementary deoxyribonucleic acid
DNA: deoxyribonucleic acid
Lcc: laccase
MCO: multi-copper oxidase
mRNA: messenger ribonucleic acid
NCBI: National Center for Biotechnological Information
NR: non-redundant
ORF: open reading frame
PCR: polymerase chain reaction
pI: isoelectric point
RNA: ribonucleic acid
RT-PCR: reverse transcriptase-polymerase chain reaction
