# Supplementary material for: De novo transcriptome assembly: a new laccase multigene family from the marine-derived basidiomycete Peniophora sp. CBMAI 1063
Source: AMB Express. 2017 Dec 20;7:222. doi: 10.1186/s13568-017-0526-7 (PMC5738328; doi:10.1186/s13568-017-0526-7)
Supplement: Supplementary file 1 — Additional file 1. Table S1. Specific primers designed for Comp15071_c0_seq5 with tails to bind amplification products in the cloning vector. Figure S1. Species distribution of all homologous unigenes. Figure S2. Gene ontology (GO) classification of assembled unigenes (Level 2). GO terms were distributed into three ontologies: molecular functions—blue bars; biological process—red bars; and cellular component—green bars. [file 13568_2017_526_MOESM1_ESM.pdf]

AMB Express

Supplementary material

De novo transcriptome assembly: a new laccase multigene family from the marine-derived basidiomycete *Peniophora* sp. CBMAI 1063

Igor Vinicius Ramos Otero[1]; Milene Ferro[2]; Maurício Bacci Jr.[1,2]; Henrique Ferreira[1]; Lara Durães Sette[1]*

[1]Departamento de Bioquímica e Microbiologia, Instituto de Biociências, Universidade Estadual Paulista Júlio de Mesquita Filho, Rio Claro, Brazil.

[2]Centro de Estudos de Insetos Sociais, Instituto de Biociências, Universidade Estadual Paulista Júlio de Mesquita Filho, Rio Claro, Brazil.

*Corresponding author at: Departamento de Bioquímica e Microbiologia - IB, Universidade Estadual Paulista Júlio de Mesquita Filho - UNESP, 24A, 1515, 13506-900, Rio Claro, SP, Brazil. Tel.: +55 19 3526-4171. larasette@rc.unesp.br (L.R. Sette)

Table S1. Specific primers designed for Comp15071_c0_seq5 with tails to bind amplification products in the cloning vector.

Primer	Sense	Sequence (5' - 3')
Lcc3 F	Foward	GGACGGGCAGGGGTAACAGGCGTTTC
Lcc3 R	Reverse	GATCCACAAAAAGCCCTTATGATGC
Lcc3B F	Foward	AAAGAATTCACCATGCGCGTTTCTGCTC
Lcc3B R	Reverse	TTTAAGCTTACGGAGTCGTGGCCATGAGCTCCATGTAGTGGTCGTAGGCGGGGCATAGGTTC

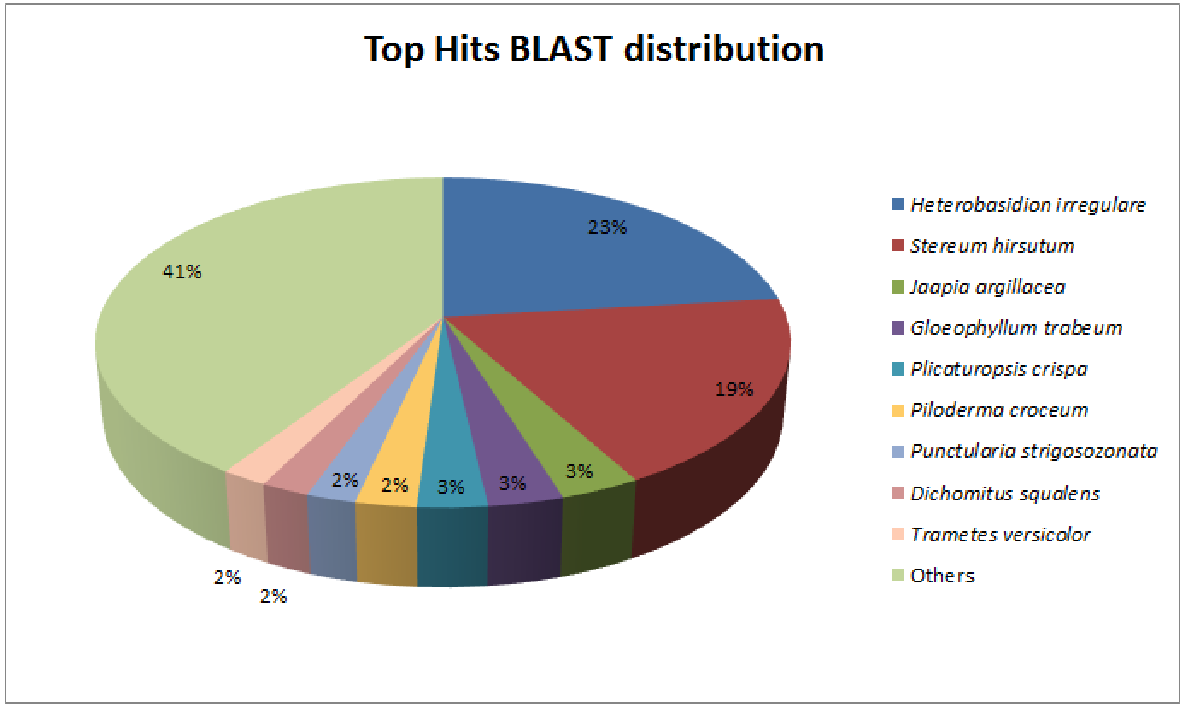

Fig S1. Species distribution of all homologous unigenes.

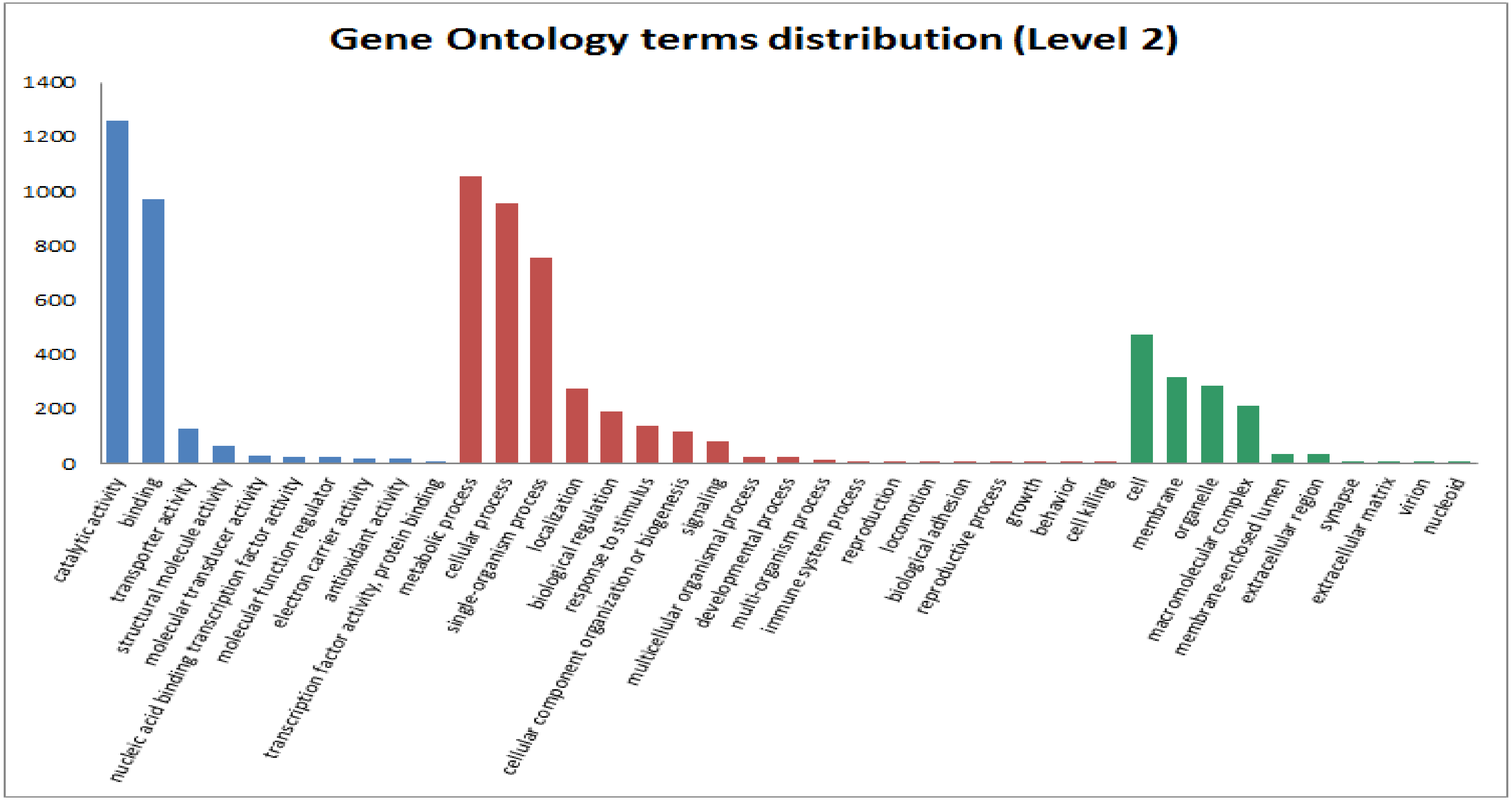

Fig S2. Gene ontology (GO) classification of assembled unigenes (Level 2). GO terms were distributed into three ontologies: molecular functions - blue bars; biological process - red bars; and cellular component - green bars.
